# A Case of Orbital Granuloma Secondary to Dermal Filler Injection

**DOI:** 10.7759/cureus.20606

**Published:** 2021-12-22

**Authors:** Stephen C Dryden, Ryan D Gabbard, Andrew G Meador, Alison E Stoner, Kimberly A Klippenstein, Ralph E Wesley

**Affiliations:** 1 Ophthalmology, Tennessee Oculoplastics, Nashville, USA; 2 Ophthalmology, Prisma Health/University of South Carolina, Columbia, USA; 3 Pathology and Laboratory Medicine, PathGroup, Brentwood, USA

**Keywords:** granulomatous reaction, transconjunctival orbitotomy, orbital mass, dermal filler, orbital granuloma

## Abstract

A foreign body granulomatous reaction is a rare complication of the subcutaneous injection of fillers. We describe a 57-year-old female that presented with a six-month history of a non-painful, right lower eyelid mass. She had a dermal filler in the zygomatic region several months before the onset of her symptoms. Anterior orbitotomy and pathology revealed multinuclear giant cells consistent with a hyaluronic acid-based filler. This case describes the clinical presentation, histopathologic features, and treatment of an orbital granuloma secondary to dermal filler injection. Our case was uncommon because the zygomatic filler migrated across the orbital septum. Additionally, transconjunctival orbitotomy was used instead of a hyaluronidase injection due to the inferior location of the granuloma.

## Introduction

Subcutaneous injection of fillers has been used as a nonsurgical treatment for facial rejuvenation. Fillers can be injected with a quick recovery and minimal discomfort making them a popular procedure for correcting soft tissue loss, rhytids, and folds. The aesthetic plastic surgery national databank estimates that over 1.3 million dermal fillers were injected in 2020 [1]. Fillers may be classified based on the duration of effect or the ability of the human body to metabolize [2]. There are many filler options available on the market and complications can arise from every type [3-10]. One such reported complication is foreign body granulomatous reaction with a reported incidence of 0.02%-0.3% depending on the type of filler injected [9,11]. The purpose of this study is to present a patient who presented with an orbital mass secondary to the migrated dermal filler with an associated granulomatous foreign body reaction. Collection and evaluation of protected patient health information for this case report were Health Insurance Portability and Accountability Act (HIPAA) compliant, and informed consent was obtained to use protected patient health information and clinical photographs. This case report also adhered to the ethical principles outlined in the Declaration of Helsinki as amended in 2013.

## Case presentation

**Figure 1:**
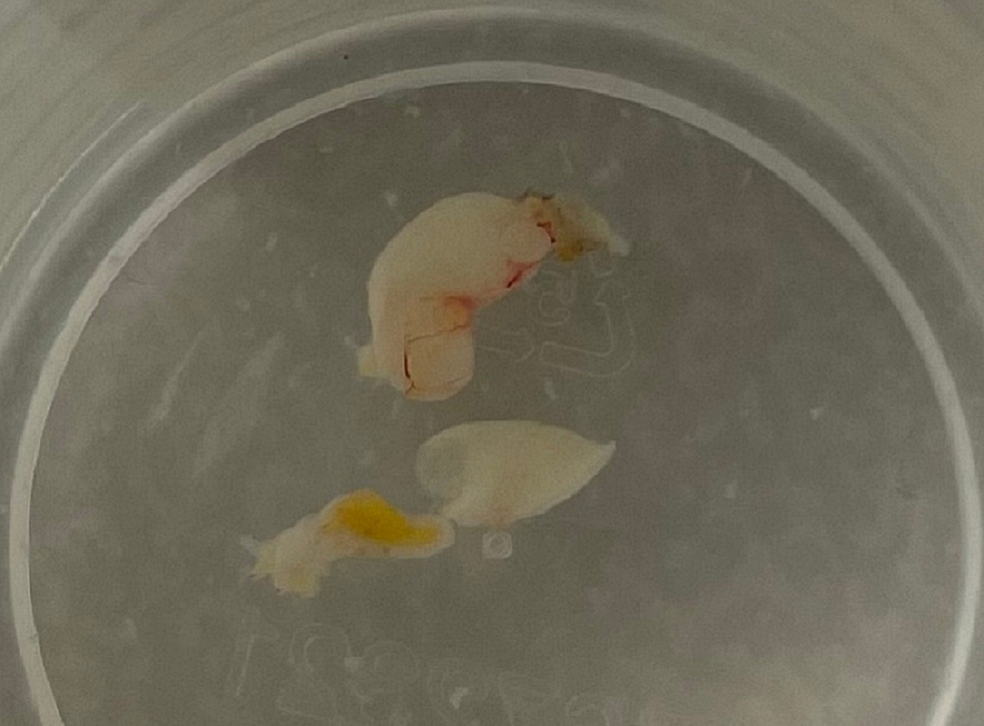
Gross view of cyst in formalin. Note grey-blue appearance with surrounding membrane with blood vessel and adipose tissue.

A 57-year-old female presented to the office for evaluation of a painless, right lower eyelid mass. The mass had been present for six months. She had a history of multiple dermal filler injections to her face over the last four years. She had a previous adverse reaction after a dermal filler full-face rejuvenation three years prior characterized by multiple periocular and perioral hard nodules that were successfully treated using oral steroids and injected hyaluronidase. Examination revealed a right lower eyelid, palpable, painless, well-circumscribed, mobile mass that appeared posterior to the orbital septum. Her visual acuity was 20/20 in the right eye with normal pupillary function, full extraocular motility, and unremarkable Hertel exophthalmometry and optic nerve function. Upon further questioning, the patient revealed that she had been injected with a Juvederm Voluma injection in the lower eyelid and lateral cheek junction in the zygomatic region to augment her cheeks approximately eight months prior to presentation. She denied any history of filler injections to her eyelid or tear troughs. She underwent a successful anterior orbitotomy through a transconjunctival incision that revealed a cystic encapsulated lesion (Figure 1).

**Figure 2:**
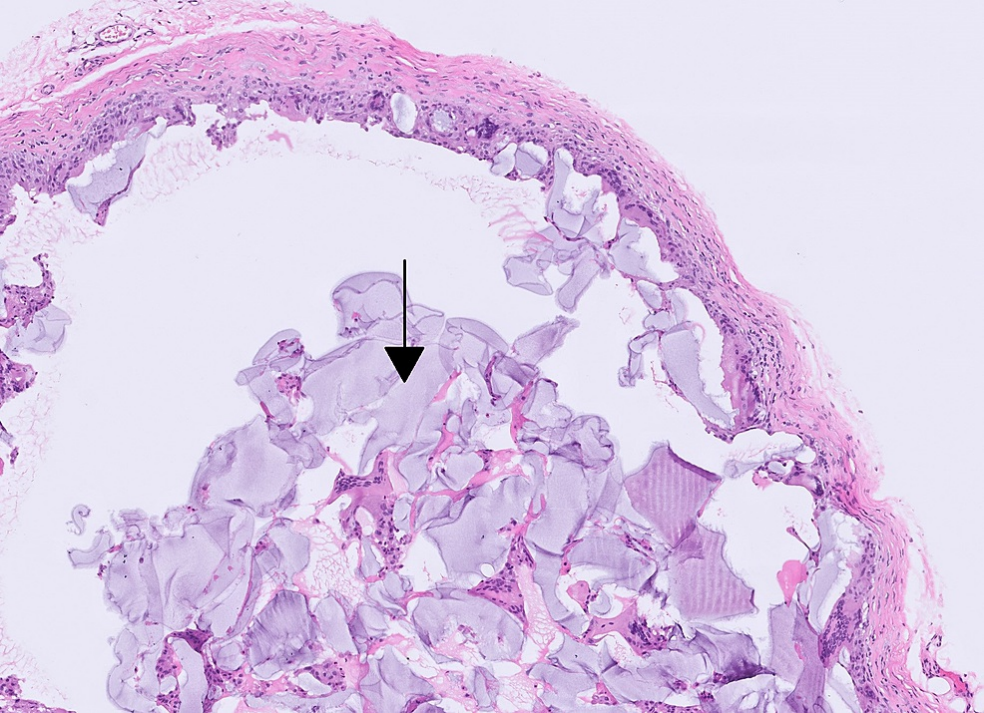
Hemotoxin and eosin stain 10 x power view of the cyst lining The cyst lining is composed of no true epithelial lining; instead, the cyst lining is entirely macrophages and a multinucleate giant cell, encased in a delicate fibrotic rim. Centrally, the acellular grey-blue material (indicated by the black arrow) is being surrounded by multinucleate foreign body giant cells.

The lesion was sent for histologic analysis, which showed acellular grey-blue material surrounded by multinucleate foreign body giant cells consistent with hyaluronic acid-based filler (Figure 2). The patient was seen at two-week, six-week, and three-month postop appointments without any complications and full resolution of symptoms.

## Discussion

Foreign body reactions occur when a material or object, such as a dermal filler originating outside the body, resists degradation by individual macrophages, which then aggregate and coalesce, leading to multinucleate giant cell formation [4]. This process may develop months to years after the injection [4,6,12]. Treatment includes local injection of steroids, hyaluronidase, and antimetabolites such as 5-Fluorouracil [4,12]. Surgical excision may be necessary for cases refractory to conservative treatment [4,12].

Hyaluronic acid (HA) is a glycosaminoglycan polysaccharide constituted from alternating residues of the monosaccharide D-glucuronic acid and N-acetyl-D-glucosamine [8]. HA-based fillers are biodegradable and have a duration of effect between six and 12 months [9]. HA occurs naturally in the body and is a component of the dermal extracellular matrix [6,8]. It is produced by smooth muscle cells, endothelial cells, adventitial cells, synovial cells, and dermal fibroblasts and plays a critical role in providing tissue support. While HA is inherent to the human body, synthetic formulations are cross-linked and therefore may cause similar foreign body reactions to our case [4,6,8].

Filler migration is described as filler at a location other than the primary injection site [2,6,12]. Filler migration may occur early or late, regardless of injector experience, technique, or type of filler injected [2,12]. Jordan et al. described several mechanisms for filler migration: injection technique-related migration, massage, muscle activity-induced displacement, gravity, lymphatic spread, and intravascular injection [2]. Foreign body reaction secondary to a migrated filler should be included in the differential diagnosis for new periorbital masses in patients with a history of dermal filler injections in areas other than their targeted injection sites. Due to the timeframe of onset of symptoms for our patient (two months after injection), injection technique, and massage are the most likely mechanisms of migration. Additionally, in regards to technique, it is generally safer to inject in the subcutaneous fat layer in comparison to the dermal fat layer. This is due to the dermal layer having a very strong immune system, making injections at this site more prone to foreign body reactions. Gravity is less likely, as her targeted injection site was in the zygomatic region, inferior to the orbit.

## Conclusions

We conclude that the evaluation and management of foreign body granulomas secondary to dermal fillers include a proper history, recognition of the substance, and imaging and/or tissue diagnosis if necessary to rule out any other sinister underlying condition in patients presenting with new orbital masses. Our case was uncommon, as it was inside the orbit, demonstrating that migrated fillers can cross the orbital septum. Most orbital granulomas secondary to fillers can be treated with injected hyaluronidase. However, our patient underwent transconjunctival orbitotomy for tissue diagnosis due to the location in the inferior orbit. Patients should be given information on the type of filler injected and counseled to seek evaluation to perform an appropriate treatment if a complication arises.

## References

[1] American Society for Aesthetic Plastic Surgery (2021). American Society for Aesthetic Plastic Surgery. Cosmetic Surgery National Data Bank statistics. https://www.surgery.org/media/statistics.

[2] Jordan DR, Stoica B (2015). Filler migration: a number of mechanisms to consider. Ophthalmic Plast Reconstr Surg.

[3] Andre P, Lowe NJ, Parc A, Clerici TH, Zimmermann U (2005). Adverse reactions to dermal fillers: a review of European experiences. J Cosmet Laser Ther.

[4] Funt D, Pavicic T (2013). Dermal fillers in aesthetics: an overview of adverse events and treatment approaches. Clin Cosmet Investig Dermatol.

[5] Iverson SM, Patel RM (2017). Dermal filler-associated malar edema: treatment of a persistent adverse effect. Orbit.

[6] Mosleh R, Mukari A, Krausz J, Hartstein ME, Azzam SH (2019). Orbit mass secondary to migration of dermal hyaluronic acid filler. JAAD Case Rep.

[7] Nathoo NA, Rasmussen S, Dolman PJ, Rossman DW (2014). Periocular mass lesions secondary to dermatologic fillers: report of 3 cases. Can J Ophthalmol.

[8] Or L, Eviatar JA, Massry GG, Bernardini FP, Hartstein ME (2017). Xanthelasma-like reaction to filler injection. Ophthalmic Plast Reconstr Surg.

[9] Parulan MA, Sundar G, Lum JH, Ramachandran U (2019). A case report on dermal filler-related periorbital granuloma formation. Orbit.

[10] Requena L, Requena C, Christensen L, Zimmermann US, Kutzner H, Cerroni L (2011). Adverse reactions to injectable soft tissue fillers. J Am Acad Dermatol.

[11] Lemperle G, Rullan PP, Gauthier-Hazan N (2006). Avoiding and treating dermal filler complications. Plast Reconstr Surg.

[12] Woodward J, Khan T, Martin J (2015). Facial filler complications. Facial Plast Surg Clin North Am.

